# Effect of deformation depth on plantar soft tissue behavior

**DOI:** 10.1186/1757-1146-7-S1-A84

**Published:** 2014-04-08

**Authors:** Jee Chin Teoh, Bena Lim, Taeyong Lee

**Affiliations:** 1 Department of Biomedical Engineering, National University of Singapore, Singapore

## Introduction

Most in vivo indentation techniques are limited by the lack of adequate indentation on the plantar tissue. Without sufficient indentation into the soft tissue, only very little and less representative information can be obtained. The purpose of this study is hence to assess the effect of deformation depth on plantar tissue behavior and to establish a set rule of optimum indentation depth that is sufficient to quantify the critical plantar soft tissue behavior.

## Methods

20 young subjects (20-25 years) participated. During the testing, the indenter [1] probed the second metatarsal head (MTH 2) and heel pad tissue with constant rate of 12mm/s. Experiment was done under load bearing (50% BW on foot tested) condition. Maximum tissue deformation induced was varied from 1.2mm to 6.0mm in steps of 1.2mm. Tissue stiffness obtained from tissue response curve was compared.

## Results

**Figure 1:**
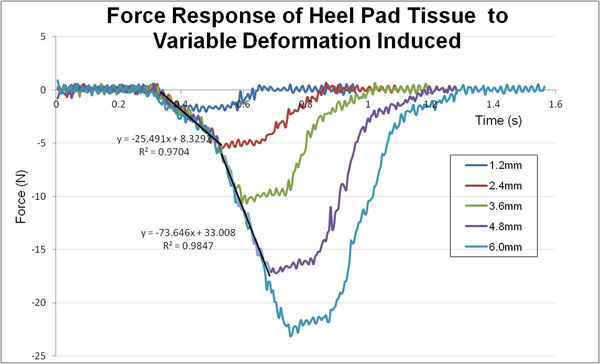
Typical force response of plantar soft tissue to the variable indentation depth.

All 20 subjects showed similar force response as demonstrated in Fig. 1, at both sites. The soft tissue response was fitted to the viscoelastic model proposed [2], represented by Equation (1).(1)

As the indenter tip goes deeper into the soft tissue beyond a threshold depth, the force gradient will increase notably. K_1_ and K_2_ depict the elastic components of soft tissue at the initial and subsequent phases of indentation. C characterizes the viscous behavior of tissue which is only prominent at the latter stage of indentation. X_s_ is the minimum indentation depth required for the tissue to exhibit nonlinear viscoelastic behavior. The parameters are listed in Table 1.

**Table 1:** Tissue properties of 2^nd^ MTH and heel

	2^nd^ MTH	Heel	p-value
**Average Tissue Thickness (mm)**	13.80 ± 1.76	18.04 ± 2.42	< 0.01*

**K_1_ (% tissue thickness /mm)**	-0.230 ± 0.122	-0.492 ± 0.151	< 0.01*

**K_2_(% tissue thickness /mm)**	-0.477 ± 0.168	-1.015 ± 0.406	< 0.01*

**X_s_ (% tissue thickness)**	16.177 ± 1.909	11.845 ± 1.284	< 0.01*

**C**	0.767 ± 0.667	1.803 ± 0.651	0.03*

*significant at 95% confidence level

## Discussion

As the indentation gets deeper, the stiffer the soft tissue becomes. We found that indentation depth which is less than the threshold depth might not be representative of the nature of plantar soft tissue. This small tissue deformation does not reflect the critical condition of soft tissue during physical activities that will expose the tissue to risk of ulceration. The threshold depth is subject dependent and is very likely to be caused by the difference in tissue composition. The next key step is to further investigate how the tissue composition will affect the threshold thickness in each subject.

The study successfully indicated the necessity to induce sufficient indentation to the soft tissue tested, in order to describe its true nature. This will eventually provide a more useful stiffness values in identification of potentially abnormal soft tissue.
